# Perplexing Tubo-Ovarian Abscess Presentation from an Inflammatory Process in a Patient with an Inconclusive Computed Tomography Scan

**DOI:** 10.7759/cureus.46760

**Published:** 2023-10-09

**Authors:** Sophia Menendez, Sydney E Moriarty, Ishan Perera, Frederic Rawlins, II

**Affiliations:** 1 Obstetrics and Gynecology, Edward Via College of Osteopathic Medicine, Blacksburg, USA; 2 Surgery, Edward Via College of Osteopathic Medicine, Blacksburg, USA; 3 Emergency Medicine, Edward Via College of Osteopathic Medicine, Blacksburg, USA

**Keywords:** percutaneous drainage, sexually transmitted infection, medical imaging, inconclusive computed tomography, tubo-ovarian abscess

## Abstract

A tubo-ovarian abscess (TOA) is an infectious mass of the adnexa. This article presents a well-documented case of a 27-year-old female presenting to the emergency department with a TOA. Physical exam findings and an initial computed tomography scan (CT) with contrast revealed a right iliopsoas abscess, an inflammatory process in the right lower quadrant, later diagnosed as a TOA with the use of ultrasound (US) without a history of sexually transmitted infection (STI). The clinical decision tree utilized in this patient’s case highlights the importance of keeping a TOA high on the list of differential diagnoses while investigating appendicitis and other inflammatory pathologies in the lower abdomen.

## Introduction

A tubo-ovarian abscess (TOA) is an infectious mass of the adnexa that may form due to complications of conditions including pelvic inflammatory disease (PID), typically due to previous sexually transmitted infections (STIs) or untreated ascending gynecological infections [1]. If left untreated, it may result in morbidities including sepsis, infertility, chronic pelvic pain, distortion of pelvic anatomy, ectopic pregnancy, and recurrent PID [1]. A TOA may present with abdominal pain, fever, nausea, vomiting, an elevated white blood cell count, and infrequently present with vaginal symptoms [2]. Literature has demonstrated that computed tomography (CT) scans with oral and intravenous (IV) contrast have improved sensitivity by 78%-100% when compared to ultrasound (US), 75%-82% [1]. Interestingly, in this case, the US provided more conclusive imaging, better discerning the pathology compared to CT scans with IV contrast. Our goal is to emphasize the importance of physical examination, the usage of appropriate imaging modalities, and the inclusion of a broad differential diagnosis when evaluating patients with non-specific gastrointestinal symptoms.

## Case presentation

History and examination

**Figure 1:**
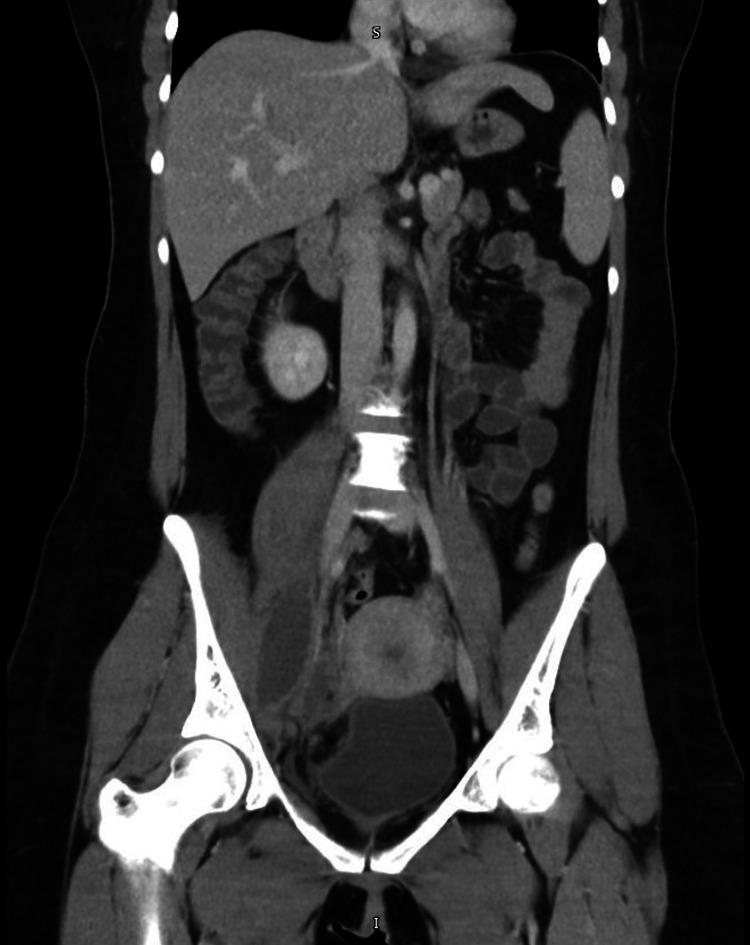
A CT scan of the abdomen and pelvis with IV contrast demonstrates a right lower quadrant inflammatory process and a right iliopsoas abscess.

A 27-year-old female, gravida 3, para 3 (G3P3), presented to the emergency department with a chief complaint of left frontal headache, nausea, vomiting, mild right lower quadrant (RLQ) pain, dizziness, and absence of appetite for three weeks. The patient denied fever and chills. On physical examination, the patient demonstrated a positive McBurney’s point and Rovsing’s sign. On pelvic examination, she had normal external female genitalia, absence of vaginal discharge, negative cervical motion tenderness, negative left adnexal tenderness to palpation, and minimal right adnexal tenderness to palpation. Laboratory studies demonstrated elevated white blood cell count 15,200/µL (normal range: 3,700 - 11,000/µL), elevated platelets 428,000/µL (normal range: 150,000 - 400,000/µL), increased neutrophil count with left shift indicating an infectious pathology 12,120/µL (normal range: 1,500 - 7,700/µL), increased monocytes 1,470/µL (normal range: 200 - 1,100/µL), elevated C-reactive protein 224.3 mg/L (normal range: <8.0 mg/L), STI polymerase chain reaction (PCR) testing for *Chlamydia trachomatis* and *Neisseria gonorrhoeae* was negative. Following a physical examination and laboratory findings, the patient underwent a CT scan of the abdomen and pelvis with IV contrast, revealing a right iliopsoas abscess with an inflammatory process in the RLQ (Figure 1).

**Figure 2:**
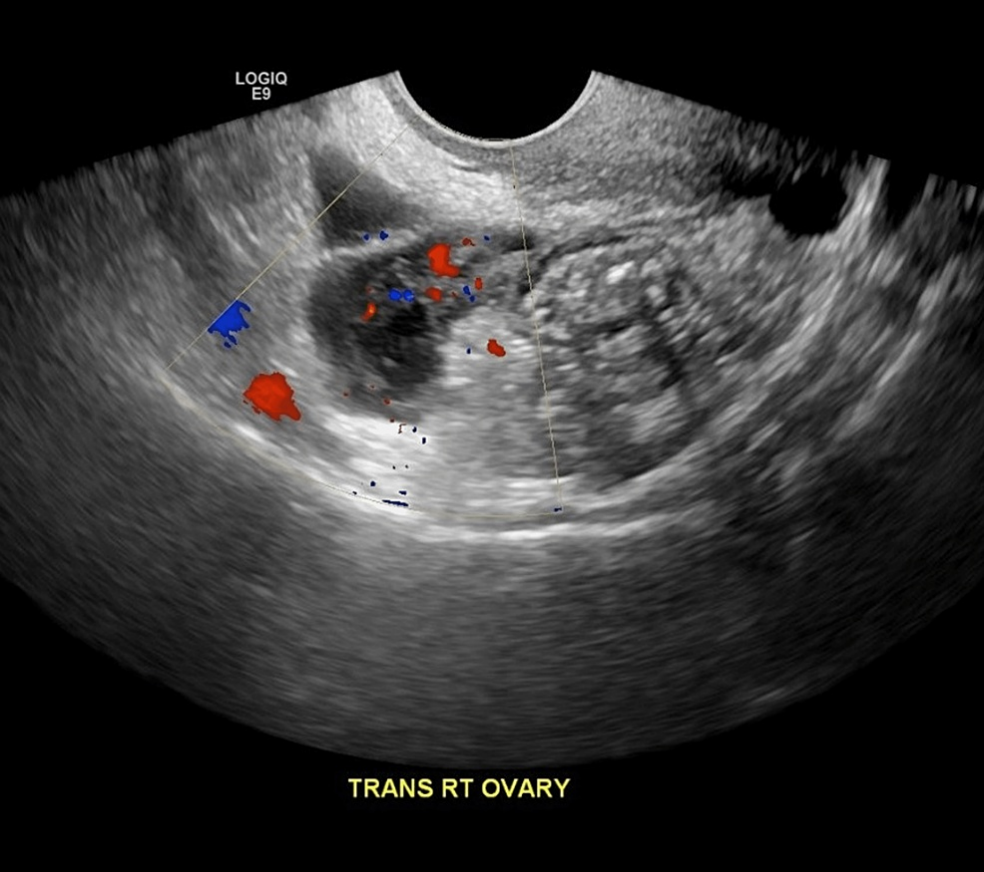
Transabdominal ultrasound demonstrates a right TOA measuring 6.2 x 3 x 2.5 cm. TOA: tubo-ovarian abscess

Following CT scan findings, the patient underwent a transabdominal US to further discern the pathological process at large. The US demonstrated a right TOA measuring 6.2 x 3 x 2.5 cm (Figure 2).

Outcome

The determination of TOA was made by the US, coupled with a physical examination. The patient was treated with an IV fluid bolus and broad-spectrum antibiotics after admission. The general surgery team was consulted and recommended percutaneous drainage of the abscess and broad-spectrum antibiotics. Interventional radiology consented to the procedure. The patient was hemodynamically stable and agreed to the treatment plan. Research demonstrates this regimen utilized in the management of patients with a TOA resulted in successful recovery in 93.4% of patients and should be considered a first-line procedure [3]. Per Reed et al., 60% of TOAs larger than 10 cm required surgery [4]. The TOA in this case measured 6.2 x 3 x 2.5 cm.

## Discussion

Observations

A suspected diagnosis of TOA is made clinically based on physical examination and confirmed via CT and US [2]. Recommendations include the use of transvaginal US to confirm suspicion, and then CT is incorporated if there is concern for malignancy or suspected gastrointestinal pathology, such as appendicitis or diverticulitis [5]. This case presented with symptoms of gastrointestinal pathology rather than common gynecologic symptoms, such as vaginal discharge or pelvic pain. This patient underwent CT, which did not clearly demonstrate a TOA but rather a potential ruptured appendicitis or other inflammatory process in the RLQ, maintaining a broad differential. Following CT, the patient underwent transabdominal US, where the TOA was confirmed. The use of US following CT was necessary to elucidate the etiology of the condition and treat the patient effectively. If the US had not been utilized, it is likely the TOA may have been missed.

Lessons

Gastrointestinal complaints are one of the most common complaints presenting to the emergency department, with an average as high as 16% presenting with the chief complaint of abdominal pain, as determined by a multicenter, dynamic cohort study of a 1.2-million-person population [6-7]. These complaints may or may not be related to the gastrointestinal system. Through examination and multiple imaging modalities, this patient was able to receive the correct diagnosis and treatment for the condition. Although CT is regarded as more sensitive for the diagnosis of conditions like TOA, the US ultimately confirmed the diagnosis. This effort by the physician to discern the root cause of the patient's symptoms demonstrates the importance of keeping a TOA high on the list of differential diagnoses, even if the patient is lacking standard gynecologic complaints or a history of STI.

## Conclusions

Our case demonstrates the necessity of keeping gynecological pathologies, such as a TOA, high on the differential diagnoses list even when patients are presenting with gastrointestinal complaints. Although research has demonstrated CT as having higher sensitivity when compared to the US, the use of both imaging modalities has a place when evaluating patients. This case specifically demonstrates the importance of a strong differential diagnosis, accompanied by strong interviewing and examination skills.
